# Normal Ovariotomy—Menstrual Physiology

**Published:** 1874-09

**Authors:** Ch. Rauschenberg

**Affiliations:** Atlanta, Ga.


### NORMAL OVARIOTOMY—MENSTRUAL PHYSIOLOGY.

ATLANTA, GA., August 17, 1874.

*Editor Atlanta Medical and Surgical Journal:*

As the report of the proceedings of the meeting of the Atlanta Academy of Medicine, of June 22, on pages 293 to 295 of your Journal of this month, was not read before the Academy at its next meeting, on account of the adjournment of the same until next October, and as this report contains only a very imperfect representation of the views expressed by me in the discussion of that evening, you are respectfully requested to admit the following additions and corrections in the September number of your Journal.

While I do not pretend to recollect the very words I used, I am confident that I made the following statements, and that the general purport of my remarks was as follows: I first requested of you to define to the Academy the indications under which you considered the operation of normal ovariotomy justifiable. I denied the intimate connection between diseases of the uterus and the ovaries, alluded to during the preceding discussion as of rather frequent occurrence, and contended that pathological conditions of either organ occurred, in the majority of instances, independent of pathological conditions of the other. I expressed the opinion that the indication for normal ovariotomy, as stated by you, to-wit: "*A desire to bring on the change of life*," with a view to cure chronic endometritis, or other serious diseases of the uterus or ovaries, was too general and indistinct. I stated that ovulation and menstruation were distinct and separate actions, performed by separate organs for different purposes; the one for the maturation and discharge of ovula, the other for the formation of a certain membrane on the walls of the uterus, for the reception and development of fecundated ova; that each one of the organs—the ovary and the uterus—performed its function independent of the other; and that while ovulation and menstruation do usually occur as coincident phenomena, each one does frequently occur by itself, at its own time, and frequently at periods of life where the other is not in operation. From these physiological reasons I denied the correctness of your position, *that removal of the ovaries would stop the menstrual flow*,* and the propriety of your operation for the purpose of bringing on this change of life, and went so far as to express my firm conviction that in less than five years this would be the accepted opinion of the medical profession. For the same reasons I expressed the opinion that removal of the ovaries could only check the specific action of that organ, be it of a normal or abnormal character, and the influence which that action might, by nerve-sympathy, exercise upon the neighboring organs, or the system generally, but could not materially affect the uterus, either in its physiological or pathological conditions.

These are, to the best of my recollection, the remarks I made on that night. If Dr. Calhoun, our reporter, failed to reproduce them in his report, I am very willing to account for it from the unusual tediousness and length of our discussion, and the great difficulty, under such circumstances, to give not too lengthy, and still a correct, statement of the many things that are said in such a discussion.

I confidently trust you will do me the justice to publish these remarks in the next number of your Journal.

Very respectfully yours,

CH. RAUSCHENBERG.

* Whether removal of the ovaries will put an end to menstruation or not depends upon the definition we give to the term "menstruation."—EDITOR.

